# Most modifiable risk factors for hamstring muscle injury in women’s elite football are extrinsic and associated with the club, the team, and the coaching staff and not the players themselves: the UEFA Women’s Elite Club Injury Study

**DOI:** 10.1007/s00167-023-07429-5

**Published:** 2023-05-01

**Authors:** Jan Ekstrand, Anna Hallén, Vittoria Marin, Håkan Gauffin

**Affiliations:** 1 Unit of Community Medicine, Department of Health, Medicine and Caring Sciences, Linköping University, Platensgatan 19, S-582 20 Linköping, Sweden; 2 Football Research Group, Linköping, Sweden; 3 Isokinetic Medical Group, FIFA Medical Centre of Excellence, London, UK; 4 Department of Orthopedics and Department of Biomedical and Clinical Sciences, Linköping University, Linköping, Sweden

**Keywords:** Soccer, Prevention, Communication, Coaching, High-speed training, Fatigue, Load

## Abstract

**Purpose:**

To describe the perceived importance of suggested hamstring injury risk factors according to chief medical officers (CMOs) of European women’s professional football clubs. A secondary objective was to compare if these perceptions differed between teams with a lower-than-average and higher-than-average hamstring injury burden.

**Methods:**

The CMOs of eleven European professional women’s football clubs were initially asked to suggest modifiable risk factors for hamstring injury. These risk factors were rated in according with their perceived importance on a 5-graded Likert scale. Participating teams were divided in two groups depending on their hamstring injury burden during the 2020/21 season. The LOW group consisted of six teams that had a lower-than-average hamstring injury burden. The HIGH group consisted of five teams that had a higher-than-average hamstring injury burden.

**Results:**

Twenty-one risk factors were suggested, most of which were extrinsic in nature, hence associated with the coaching staff, the team or the club organization rather than with the players themselves. The risk factors with the highest average importance were: “lack of communication between medical staff and coaching staff” and “load on players” (each with a weighted average of 3.9), followed by “lack of regular exposure to high-speed football actions during training” and “playing matches 2–3 times a week” (weighted average of 3.8 and 3.7). Differently from the LOW group, the HIGH group perceived the coaching factors (style of coach leadership, training/exercise surveillance by coaching staff) as more important.

**Conclusion:**

In accordance to the eleven CMOs recruited in this study, most risk factors for hamstring injuries are extrinsic in nature and associated with the club, the team, and the coaching staff, and not the players themselves.

**Level of evidence:**

Level III.

## Introduction

Hamstring injury has been reported to be the most common [20, 31] injury subtype in women’s elite-level football players, constituting 12–16% of all time-loss injuries [20, 24, 31]. Previous studies suggested that a team with a 19–22-player squad could typically expect three to four hamstring injuries each season [20, 24].

Various risk factors for hamstring injuries have been proposed [17, 31], and clubs have been trying to embrace systematic hamstring prevention programs [3]. Older age and a history of hamstring injury are reported as being the strongest risk factors for hamstring injuries [17, 21], but these are both non-modifiable risk factors. Player factors, such as hamstring muscle strength, muscle imbalance, and flexibility, have been investigated as modifiable risk factors but with contradictory results [17, 21].

In 2017, the Union of European Football Associations (UEFA) initiated a research project aimed to reduce injuries and increase player safety in women’s professional football—the Women Elite Club Injury Study (WECIS).

The WECIS has been carried out by the international research team called Football Research Group (FRG). In collaboration with UEFA, FRG has since 2001 continuously carried out an injury surveillance study on male elite football the UEFA Elite Club Injury Study (ECIS) [5, 8, 9, 12, 37]. During the 2017–2018 season, FRG started a pilot study on women’s elite clubs in Europe, using the same validated methodology as in the ECIS [5, 8, 9, 12, 37]. Thirteen teams participated in the pilot study, twelve of them participating in the UWCL (UEFA Women’s Champions League) and eleven of these 12 qualified for the round of 32 that season. Based on the positive results from the pilot study, UEFA decided to initiate and fund a continuous Women’s Elite Club Injury Study (WECIS) starting from season 2018–19. For continuity reasons, UEFA has decided that teams that participated in the pilot study should be invited to continue participating.

The aim of this study was to assess the educated opinions on preventable risk factors for hamstring injuries. Results are based on information received from eleven Chief Medical Officers (CMOs) of European women’s elite clubs. A second aim was to compare the opinions received from teams that had lower-than-average hamstring injury rates with teams that had higher-than-average hamstring injury rates during the season 2020/21.

Based on previous studies [20, 24], it was hypothesized that individual player factors, such as lack of eccentric hamstring muscle strength, would have been perceived as the most important modifiable risk factor for hamstring injuries.

## Materials and methods

### Study design

This was a questionnaire study to assemble the opinions of CMOs of clubs that participated in WECIS season 2020–21.

### Inclusion criteria for study participants

This is a sub-study of the WECIS carried out during the season 2020–21. We only included teams that provided complete injury data throughout the entire season and answered the questionnaires. Eleven teams from seven countries participated to the study as they provided data and answers requested (FC Barcelona and Club Atlético Madrid from Spain, Chelsea FC and Manchester City from England, AFC Ajax and PSV Eindhoven from the Netherlands, Juventus FC and AC Milan from Italy, FC Bayern München from Germany, Olympique Lyonnais from France, and Sporting Club de Portugal).

### Exposure and injury data collection

Exposure and injury data collection for the season 2020/21 was completed in September 2021. Definitions and method of data collection have been previously described in details [1, 16, 19]. Injury was defined as: “any physical complaint sustained by a player resulting from a football match or football training that leads to the player being unable to fully take part in football training or match play thereafter”. A hamstring injury was defined as: “a traumatic distraction or gradual onset injury to the hamstring muscle group”. Injury burden was defined as: “number of lay-off days per 1000 player hours ((Σ lay-off days/Σ exposure hours) × 1000)”. Injury incidence was defined as: “ number of injuries per 1000 player hours ((Σ injuries/Σ exposure hours) × 1000)”.

The 11 teams were divided into two groups; one group with 6 teams having lower-than-average hamstring injury burden compared to all teams (LOW group), and one group including 5 teams (HIGH group) with higher-than-average hamstring injuries burden during the 2020/21 season.

Table 1 shows the hamstring injury data for all the 11 clubs as well as the teams in the two groups.

**Table 1:** Hamstring injury data of clubs with lower, average or higher hamstring injury rates

	Group LOW teams with lower-than-average HI burden (*n* = 6)	Group HIGH Teams with higher-than-average HI burden (*n* = 5)	All teams (*N* = 11)
Hamstring injury burden season 2020/21	7	19	14
Incidence hamstring injury during match play season 2020/21	1.0	4.2	2.6
Number of days absent due to hamstring injuries season 2020/21	45	126	82

Injury was defined as: “any physical complaint sustained by a player resulting from a football match or football training that leads to the player being unable to fully take part in football training or match play thereafter”. A hamstring injury was defined as: “a traumatic distraction or gradual onset injury to the hamstring muscle group”. Injury burden was defined as: “number of lay-off days per 1000 player hours ((Σ lay-off days/Σ exposure hours) × 1000)”. Injury incidence was defined as: “ number of injuries per 1000 player hours ((Σ injuries/Σ exposure hours) × 1000)”

### The survey questionnaire

During the period May to November 2022, the CMOs of the eleven clubs eligible for inclusion were invited to participate in the questionnaire study. All eleven accepted.

The CMOs were informed that the aim of the questionnaire was to assemble and evaluate their expert opinions and conclusions on preventable risk factors for hamstring injury in women’s elite football. The medical officers were asked to base their answers on their practice and experience throughout the season 2020/21. After agreeing to participate in the study, they were provided with access to the questionnaire using the online survey software SurveyMonkey (SurveyMonkey^©^, California, USA). The survey was conducted in two stages. In the first stage, the participants were asked to provide their opinions on 21 modifiable risk factors for hamstring injuries, which previously were suggested by CMOs in male teams [6]. The CMOs of women’s teams were also asked to propose additional risk factors, which haven’t been previously identified; however, no such suggestions were forwarded. In the second stage, CMOs used a 5-garde Likert scale (very important, important, moderate importance, minor importance, and no importance) to evaluate their perceived importance of each 21 modified risk factor.

### Survey analysis

Scores were as follows: Very important was weighted 4; Important = 3; Moderate importance = 2; Minor importance = 1; and No importance = 0. A “No opinion” alternative was also available. As a result, scores were averaged, and risk factors ranked in order of averages [19, 20]. For each risk factor, a weighted average was calculated:

$$\Sigma \left( {{\text{variables }}*{\text{ weight}}} \right)/(\Sigma \,{\text{all weights}})$$

### Patient and public involvement

This research was carried out without patient (player) involvement i.e., players were not invited to comment on the study design or to contribute to the drafting of this document.

## Results

Eleven CMOs replied to the survey. Perceived risk factors, their importance, and their weighted average are shown in Table 2.

**Table 2:** Perceived modifiable risk factors for hamstring injuries, divided between intrinsic and extrinsic factors

Intrinsic risk factors (players)	Total group (*n* = 1) weighted average (max = 4)	Group LOW (*n* = 6) Weighted average (max = 4)	Group HIGH (*n* = 5) Weighted average (max = 4)
Lack of eccentric strength of the hamstrings	3.5	3.5	3.4
Residual weakness after previous hamstring injury	3.4	3.3	3.4
Fatigue	3.2	3.3	3.0
Poor core stability (lumbo-pelvic control)	3.1	3.2	2.8
Strength asymmetry of hamstrings (R/L ratio, Q/H ratio)	3.0	2.7	3.6
Poor coordination	2.8	2.5	3.2
Player wellness (sleep patterns, relationships, etc.)	2.7	2.8	2.4
Poor flexibility	2.5	2.3	2.6
Poor nutrition	2.3	2.3	2.3
**Mean**	**26.5/9 = 2.9**	**25.9/9 = 2.9**	**26.7/9 = 3.0**
Extrinsic risk factors (coaching, team, club)			
Lack of communication between medical staff and coaching staff	3.9	3.9	3.9
Load on players	3.9	3.8	4.0
Lack of regular exposure to high-speed football during training	3.8	3.8	3.8
Playing matches 2–3 times a week	3.7	2.8	4.0
Lack of regular exposure to strength training (eccentric/isometric/concentric)	3.5	3.3	3.8
Lack of in-season recovery strategies	3.2	3.2	3.2
Lack of interest in prevention strategies in the team or club	3.2	3.2	3.2
Off-season loading/recovery	2.8	2.8	2.8
Number and expertise of the medical staff	2.8	2.8	2.8
Style of coach leadership	2.6	2.3	3.0
Training/exercise surveillance by coaching staff	2.4	1.3	3.0
Medical budget	1.9	2.2	1.8
**Mean**	**37.7/12 = 3.1**	**35.4/12 = 3.0**	**39.3/12 = 3.3**

Among the 21 perceived modifiable risk factors, 12 were considered extrinsic factors (relating to coaching, team, and club) and 9 suggested being intrinsic or player factors. Further, the importance of the factors, as expressed as the mean of the weighted averages, was 3.1 for the extrinsic factors compared to 2.9 for the intrinsic factors. A “lack of communication between the medical staff and the coaching staff” and “load on players” were perceived as the most important or second most important risk factor in both subgroups.

The greatest difference between the two groups was the perception of coach actions (coach leadership and coach surveillance of training/exercises) as well as “playing matches 2–3 times a week”.

The group with higher hamstring injury rates (the HIGH group) perceived these factors as more important compared to the teams with less hamstring injuries (LOW group). The HIGH group also perceived the player factors “strength asymmetry” and “poor coordination” as more important compared to the LOW group.

## Discussion

The most important finding was that the CMOs included in the study felt that extrinsic (coaching, team, and club) preventable risk factors were the most important compared to the intrinsic ones (players). “Lack of communication between medical staff and coaching staff”, “load on players”, “lack of regular exposure to high-speed football during training”, and “playing matches 2–3 times a week” were the risk factors with the highest average importance. The greatest differences between the two groups were the perception of the coach actions (coach leadership and coach surveillance of training/exercises) as well as “playing matches 2–3 times a week”.

Hamstring injury is the most common injury diagnosis in women’s professional football [20, 31], and thus, exceedingly important to prevent. Several risk factors may contribute to hamstring injuries. Fatigue is regarded as a relevant risk factor behind the majority of hamstring injuries [3, 7, 21, 22, 33, 35, 37]. The coaches control numerous variables that may lead to fatigue—excessive training, too many matches, muscle overload, or poor training periodization leading to under-training and muscular dysfunction. An ECIS study showed that muscle injuries occur more frequently toward the end of a match [7, 9] or during/after a congested match period with little time for recovery [2]. All these factors are potentially modifiable.

Hamstring injuries mostly occur during sprinting and other high-velocity actions [18]. Therefore, regular and consistent exposure to high-speed football actions prepares the hamstrings for similar moves occurring during games [27, 30]. Lack of sufficient high-speed play during training increases the risk for hamstring injury during the competition. Training should mimic match play to adapt the muscles to the game demand [18], also this factor is mainly controlled by coaches.

There are several well-designed controlled studies showing that the Nordic hamstrings exercise (NHE) program effectively reduces hamstrings injuries [3, 34]. In addition, systematic reviews and meta-analysis reports a preventive effect [36]. Elite male teams that implemented NHE during training for most of the players have been shown to sustain fewer hamstring injuries than teams that only used the NHE for individual players [6]. Existing literature indicates that the NHE may be effective in reducing the number of hamstring injuries in football. However, for a preventive measure to be effective, it is important that players, coaches, and officials are motivated to implement the program [15]. Once again, the importance of coaches is empathized.

Risk factors found in male elite football may differ in women’s football. In fact, distinct dissimilarities have been shown in male and women football injuries [20, 25, 26]. In addition, females are significantly underrepresented in sport research [4, 28]. There are different hormonal characteristics in women compared to men. Men have 15- to 20-fold greater circulating testosterone than women which influences performance [23], but also possibly affects the injury panorama. Differences in psychological factors may also have an impact [29]. Those considerations call for further studies on elite women athletes, introducing different methodological approaches [14].

The greatest difference between the two groups in the present study was the perception of coaches’ actions (coach leadership and coach surveillance of training/exercises). There is an association between overall injury rate (not specifically hamstring injuries) and the leadership style of the main coach [11]. Surveillance and training/exercise execution correction was included as one of the seven preventative measures in the study by Ekstrand et al. already in 1983; first, RCT showing that it is possible to prevent sports injuries [11].

For a preventive measure to be successful, it is important that players, coaches, and officials are motivated to use the programs [32]. There is an association between overall injury rates (not specifically hamstrings injuries) and the quality of internal communication at the club [10]. A previous study has highlighted the responsibility of the club and coaching staff in reducing the risk of hamstring injury specifically, in men’s professional football [13].

The main strength of this survey is that it includes the opinions of medical experts from elite clubs.

The study has limitations. First, hamstring injury, according to our inclusion criteria, comprises a heterogeneous group including structural (partial or total muscle fibers ruptures) and functional (no macroscopic muscle fibers disruption) injuries, with different foci and severities. Second, there may be different causes for acute or gradual onset hamstring injuries. This was not considered in the present study. The study is also limited by a relatively small sample size and a short observation period. It would have been more beneficial with a prospective study extending over several consecutive seasons, to establish a more robust association between potential hamstring risk factors and hamstring injury rates. Such a study would be less likely to be influenced by flukes in injury rates and changes in training practice. The generalizability of the results from these six successful clubs to other high-level or semi-professional clubs is unclear. Finally, as this is a descriptive study, we cannot infer any causality between expert opinions on risk factors and injury rates since the clubs may have differed regarding other risk factors for hamstring injury, or confounding factors unknown to us.

This study highlights the responsibility of the club and coaching staff in reducing the risk for hamstring injury in women’s professional football. A better understanding of the importance of communication between medical and coaching staff, as well as improvement in load management and training content during the football season could potentially lead to a fall in the rate of hamstring injuries among women professional players.

## Conclusions

This study highlights the responsibility of the club and coaching staff in reducing the hamstring injury risk in women professional football. According to the eleven CMOs recruited in this study, most risk factors for hamstring injuries are extrinsic and associated with the club and coaching staff, and not the players themselves.

## Data Availability

The dataset from this study is held securely in coded form and data are not available to the public.
